# A new spider species of the *Pholcusyichengicus* species group (Araneae, Pholcidae) from Hebei Province, northern China

**DOI:** 10.3897/BDJ.12.e141640

**Published:** 2024-12-05

**Authors:** Jinglin Li, Xiaoqing Zhang, Zhiyuan Yao

**Affiliations:** 1 College of Life Science, Shenyang Normal University, Shenyang 110034, Liaoning, China College of Life Science, Shenyang Normal University, Shenyang 110034 Liaoning China

**Keywords:** biodiversity, cellar spiders, invertebrate, morphology, taxonomy

## Abstract

**Background:**

The *Pholcusyichengicus* species group currently contains 47 species. It is mainly distributed in central and southern China, as well as northern Thailand.

**New information:**

*Pholcuschangli* Yao, sp. nov. is described as a new species of the *P.yichengicus* species group collected from northern China (Hebei Province).

## Introduction

**Figure 1.**
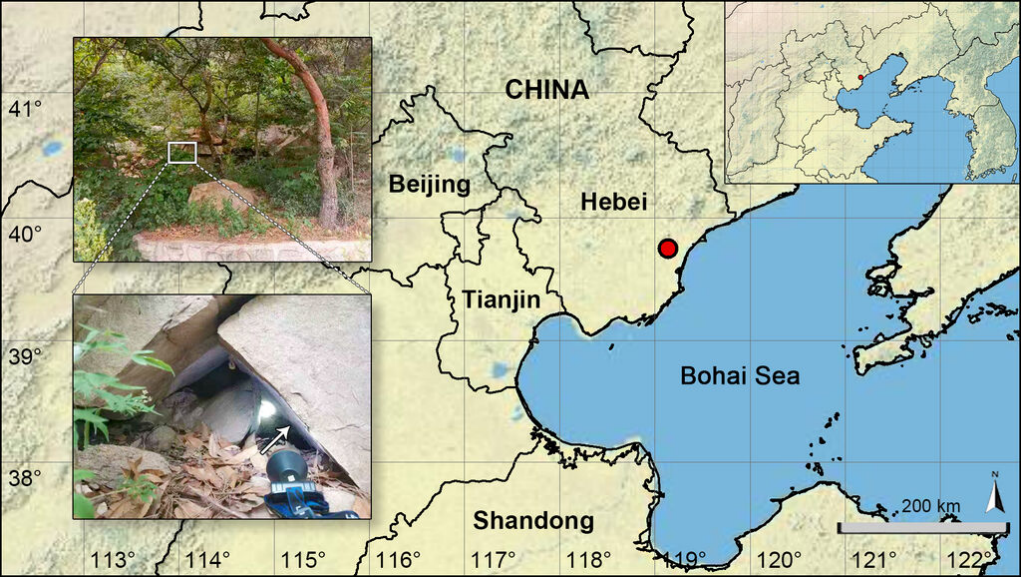
Distribution record of *Pholcuschangli* sp. nov. in Hebei Province, China. Arrow in the insert points at habitat.

*Pholcus* Walckenaer, 1805 is the most diverse genus in Pholcidae C.L. Koch, 1850, with 408 species belonging to 21 species groups (Huber 2011, Huber et al. 2018, WSC 2024). Currently, the *Pholcusyichengicus* species group contains 47 species, 45 of them being distributed in central and southern China, as well as northern Thailand (e.g. Huber (2011), Yao and Li (2012), Zhu et al. (2018), Yang et al. (2024a), Yang et al. (2024b)). Two species, *P.guani* Song & Ren, 1994 and *P.bajia* Lu, Yao & He, 2022, are known from northern China (Song and Ren 1994, Lu et al. 2022a). In this paper, we describe a new species of the *P.yichengicus* species group, based on material of both sexes collected in Hebei Province, northern China (Fig. 1).

## Materials and methods

Specimens were examined and measured with a Leica M205 C stereomicroscope. The male left palp was photographed. The epigyne was photographed before dissection. The vulva was photographed after treating it in a 10% warm solution of potassium hydroxide (KOH) to dissolve soft tissues. Images were captured with a Canon EOS 750D wide zoom digital camera (24.2 megapixels) mounted on the stereomicroscope mentioned above and assembled using Helicon Focus v. 3.10.3 image stacking software (Khmelik et al. 2005). All measurements are given in millimetres (mm). Leg measurements are shown as: total length (femur, patella, tibia, metatarsus, tarsus). Leg segments were measured on their dorsal side. The distribution map was generated with ArcGIS v. 10.2 (ESRI Inc.). The specimens studied are preserved in 75% ethanol and deposited in the College of Life Science, Shenyang Normal University (SYNU) in Liaoning, China.

Terminology and taxonomic descriptions follow Huber (2011), Yao et al. (2021) and Lu et al. (2022b). The following abbreviations are used: **a** = appendix, **aa** = anterior arch, **ALE** = anterior lateral eye, **AME** = anterior median eye, **b** = bulb, **da** = distal apophysis, **e** = embolus, **fa** = frontal apophysis, **kn** = knob, **L/d** = length/diameter, **pa** = proximo-lateral apophysis, **PME** = posterior median eye, **pp** = pore plate, **pr** = procursus, **u** = uncus.

## Taxon treatments

### Pholcus changli

Yao sp. nov.

7F8B9282-62E4-5567-B09D-C975772FCF50

04FC002B-A96A-4019-8832-AA07C73D0ED3

#### Materials

- **Type status:** Holotype. **Occurrence:** recordedBy: Zhiyuan Yao, Jinglin Li and Meichen Yan; individualCount: 1; sex: male; lifeStage: adult; occurrenceID: D9CE08FE-6B45-5A66-B374-0D7C19672401; **Taxon:** order: Araneae; family: Pholcidae; genus: Pholcus; **Location:** country: China; stateProvince: Hebei; municipality: Qinhuangdao; locality: Changli County; verbatimLocality: Jieshishan Scenic Spot; verbatimElevation: 117 m a.s.l.; verbatimLatitude: 39°44.72'N; verbatimLongitude: 119°8.61'E; **Event:** samplingProtocol: by hand; year: 2024; month: 7; day: 20; **Record Level:** institutionCode: SYNU-Ar00443
- **Type status:** Paratype. **Occurrence:** recordedBy: Zhiyuan Yao, Jinglin Li and Meichen Yan; individualCount: 1; sex: female; lifeStage: adult; occurrenceID: B3E810DE-1AC9-5713-9992-6BDF9C89511D; **Taxon:** order: Araneae; family: Pholcidae; genus: Pholcus; **Location:** country: China; stateProvince: Hebei; municipality: Qinhuangdao; locality: Changli County; verbatimLocality: Jieshishan Scenic Spot; verbatimElevation: 117 m a.s.l.; verbatimLatitude: 39°44.72'N; verbatimLongitude: 119°8.61'E; **Event:** samplingProtocol: by hand; year: 2024; month: 7; day: 20; **Record Level:** institutionCode: SYNU-Ar00444

#### Description

**Figure 2.**
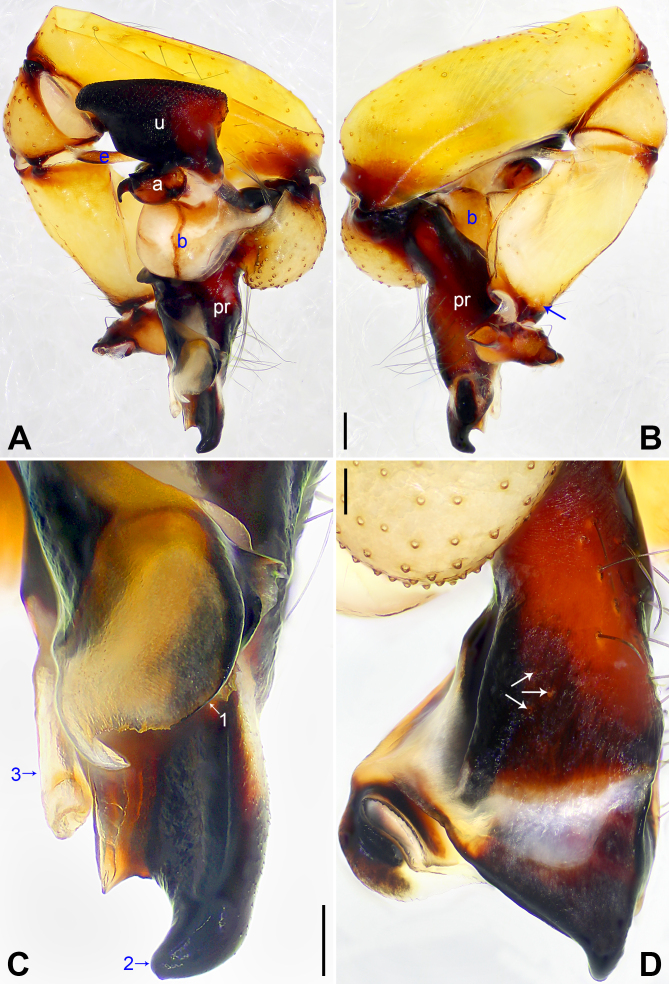
*Pholcuschangli* sp. nov., holotype male. **A, B** Palp (**A** Prolateral view; **B** Retrolateral view, arrow points at retrolatero-proximal protrusion); **C, D** Distal part of procursus (**C** Prolateral view, arrow 1 points at prolatero-subdistal apophysis, arrow 2 points at dorso-distal apophysis, arrow 3 points at ventro-subdistal membranous process; **D** Dorsal view, arrows point at dorsal spines). Abbreviations: a = appendix, b = bulb, e = embolus, pr = procursus, u = uncus. Scale bars: 0.20 mm (**A, B**); 0.10 mm (**C, D**).

**Figure 3.**
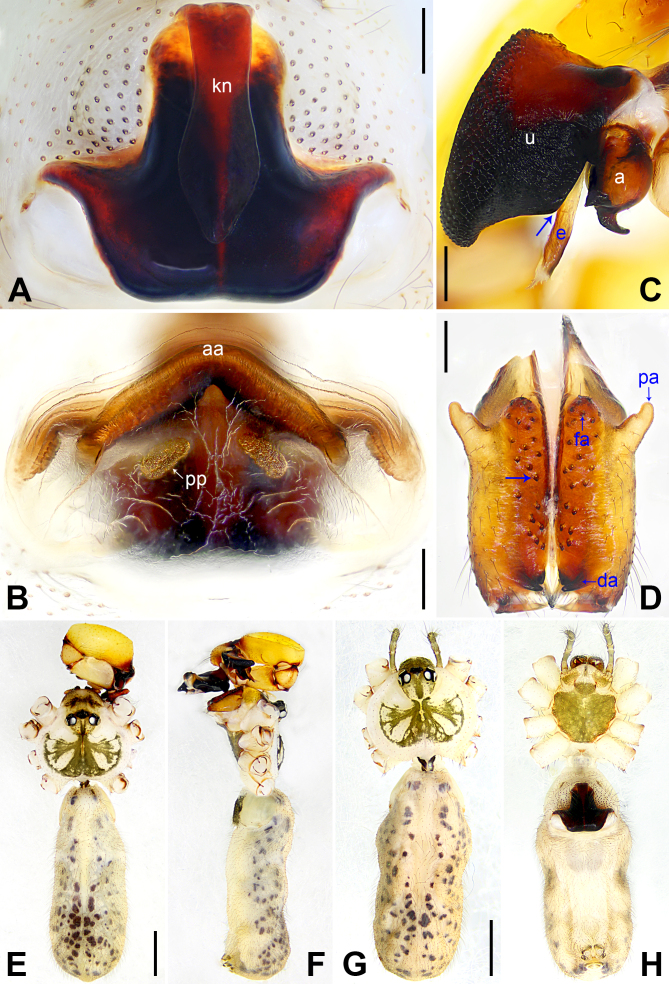
*Pholcuschangli* sp. nov., holotype male (**C–F**) and paratype female (**A, B, G, H**). **A** Epigyne, ventral view; **B** Vulva, dorsal view; **C** Bulbal apophyses, prolateral view, arrow points at latero-median protrusion; **D** Chelicerae, frontal view, arrow points at median cones; **E–H** Habitus (**E, G** Dorsal view; **F** Lateral view; **H** Ventral view). Abbreviations: a = appendix, aa = anterior arch, da = distal apophysis, e = embolus, fa = frontal apophysis, kn = knob, pa = proximo-lateral apophysis, pp = pore plate, u = uncus. Scale bars: 0.20 mm (**A–D**); 1.00 mm (**E–H**).

**Male** (holotype): Total length 6.00 (6.28 with clypeus), carapace 1.70 long, 2.13 wide, opisthosoma 4.30 long, 1.72 wide. Leg I: 52.33 (12.82, 0.86, 13.14, 22.37, 3.14), leg II: 32.63 (9.20, 0.79, 8.08, 12.76, 1.80), leg III: 23.44 (6.79, 0.75, 5.51, 9.04, 1.35), leg IV: 31.66 (9.17, 0.75, 7.90, 12.18, 1.66); tibia I L/d: 69. Eye interdistances and diameters: PME–PME 0.30, PME 0.15, PME–ALE 0.05, AME–AME 0.05, AME 0.11. Sternum width/length: 1.27/1.09. Habitus as in Fig. 3E and F. Carapace yellowish, with brown radiating marks and marginal brown bands; ocular area yellowish, with median and lateral brown bands; clypeus yellowish, with brown marks; sternum brown. Legs yellowish, but dark brown on patellae and whitish on distal parts of femora and tibiae, with darker rings on subdistal parts of femora and proximal and subdistal parts of tibiae. Opisthosoma yellowish, with dorsal and lateral brown spots. Chelicerae with pair of proximo-lateral apophyses (pa in Fig. 3D), pair of distal apophyses (da in Fig. 3D) without teeth, pair of frontal apophyses (fa in Fig. 3D) and several small median cones (arrow in Fig. 3D). Palps as in Fig. 2A and B; trochanter with retrolaterally strongly bulged ventral apophysis; femur with retrolatero-proximal protrusion (arrow in Fig. 2B) and indistinct ventral protrusion; tibia with prolatero-ventral protrusion; procursus simple proximally, but complex distally, with prolatero-subdistal apophysis (arrow 1 in Fig. 2C) bearing proximally membranous part, curved dorso-distal apophysis (arrow 2 in Fig. 2C), ventro-subdistal membranous process (arrow 3 in Fig. 2C) and three dorsal spines (arrows in Fig. 2D); uncus with scales and latero-median protrusion (arrow in Fig. 3C); appendix hooked, with swollen subdistal branch (a in Fig. 3C); embolus slightly sclerotised, with transparent distal projections (e in Fig. 3C). Retrolateral trichobothrium on tibia I at 5% proximally; legs with short vertical setae on tibiae, metatarsi and tarsi; tarsus I with 33 distinct pseudosegments.

**Female** (paratype, SYNU-Ar00444): Similar to male, habitus as in Fig. 3G and H. Total length 5.66 (5.85 with clypeus), carapace 1.66 long, 2.00 wide, opisthosoma 4.00 long, 1.60 wide; tibia I: 9.25; tibia I L/d: 46. Eye interdistances and diameters: PME–PME 0.26, PME 0.16, PME–ALE 0.05, AME–AME 0.06, AME 0.08. Sternum width/length: 1.16/0.80. Clypeus brown. Epigyne strongly sclerotised, anchor-shaped, with wedge-shaped knob (kn in Fig. 3A). Vulva with curved, dorsally sclerotised and ventrally membranous anterior arch (aa in Fig. 3B) and pair of nearly elliptic pore plates (3× longer than wide, pp in Fig. 3B).

#### Diagnosis

The species resembles *P.bajia* Lu, Yao & He, 2022 (Lu et al. 2022a: 4, figs. 2A–D and 3A–H) by having similar male chelicerae (Fig. 3D), but can be distinguished by dorso-distal apophysis of procursus distally curved (arrow 2 in Fig. 2C vs. distally straight), by uncus latero-medially protruding (arrow in Fig. 3C vs. prolatero-medially protruding), by appendix with sclerotised subdistal branch (a in Fig. 3C vs. membranous subdistal branch), by epigynal knob approximately 4/5 length of epigynal plate (kn in Fig. 3A vs. 1/2) and by vulval pore plates 3× longer than wide (pp in Fig. 3B vs. 1.5×).

#### Etymology

The specific name refers to the type locality; noun in apposition.

#### Distribution

China (Hebei, type locality; Fig. 1).

#### Biology

Underside of overhang on rocky cliffs in the mountain area (Fig. 1).

## Supplementary Material

XML Treatment for Pholcus changli
